# Evidence-based preprocessing improvement bundle for surgical instruments: reducing surgical infection risks via quality improvement

**DOI:** 10.3389/fpubh.2026.1930642

**Published:** 2026-10-27

**Authors:** Ruixue Hu, Wei Pan, Liangying Yi, Juan Hu, Hongxia Zhang, Yanhua Chen

**Affiliations:** 1 Department of Sterile Processing Nursing, West China Second University Hospital, Sichuan University, Chengdu, Sichuan, China; 2 West China School of Nursing, Sichuan University, Chengdu, Sichuan, China; 3 Key Laboratory of Birth Defects and Related Diseases of Women and Children, Ministry of Education, Sichuan University, Chengdu, Sichuan, China

**Keywords:** central sterile supply department, cleaning, evidence-based practice, preprocessing, surgical instruments

## Abstract

**Introduction:**

Inadequate preprocessing of surgical instruments can leave biofilm residue, creating potential risks during instrument reprocessing and increasing the risk of healthcare-associated infections, thereby compromising patient safety and healthcare quality. This study aimed to develop a standardized surgical instrument preprocessing protocol based on evidence-based practice and validate its effectiveness in improving instrument preprocessing quality and central sterile supply department (CSSD) staff’s knowledge of nosocomial infection prevention and control and increasing their compliance with preprocessing criteria.

**Methods:**

Guided by the Joanna Briggs Institute (JBI) model of evidence-based healthcare, this study summarized the best evidence for surgical instrument preprocessing, analyzed implementation barriers in clinical practice, and formulated standardized audit criteria and intervention strategies for surgical instrument preprocessing. A one-group pretest-posttest study was conducted to assess compliance with audit criteria in preprocessing procedures, rate of surgical instruments returned to the decontamination area for initial re-cleaning, and CSSD staff’s knowledge of nosocomial infection prevention and control.

**Results:**

After applying the evidence in practice, 10 of 20 audit criteria achieved a 100% compliance rate. The rate of surgical instruments returned to the decontamination area for initial re-cleaning dropped from 0.67 to 0.085% (χ^2^ = 3512.527; *p* < 0.001; absolute risk difference = 0.58%, with 95% confidence interval (CI) of 0.56–0.60%; risk ratio = 7.93, with a 95% CI of 7.31–8.60). CSSD staff’s mean scores for knowledge of nosocomial infection prevention and control increased from 58.06 ± 9.097 to 95.83 ± 3.930 (*t* = 22.520, *p* < 0.001).

**Conclusion:**

The intervention, based on the JBI evidence-based model for improving the quality of surgical instrument preprocessing, could standardize instrument preprocessing procedures, reduce the proportion of surgical instruments returned to the decontamination area for initial re-cleaning, and improve CSSD staff’s theoretical knowledge of instrument preprocessing. This study can provide evidence to support quality control in surgical instrument preprocessing and contribute to the prevention and control of nosocomial infections.

## 1 Introduction

Surgical instrument contamination and biofilm residue are major risk factors for healthcare-associated infections, posing a threat to patient safety. Inadequate or nonstandard preprocessing of reusable surgical instruments can lead to persistent microbial residue and incomplete sterilization, thereby increasing the risk of postoperative infections and affecting the overall quality of public healthcare services (1). Surgical instrument preprocessing is critical for instrument cleaning. It refers to the initial reprocessing of devices, utensils, and articles after use, including removing visible contaminants from instrument surfaces, keeping instruments moist, and protecting sharp and delicate instruments from damage. It includes two stages: point-of-use preprocessing in the operating room before instruments are transported to the Central Sterile Supply Department (CSSD), and preprocessing after instruments are delivered to the CSSD but before they are cleaned there (2). The results of Weber et al. showed that preprocessing surgical instruments immediately after use during surgery and at the end of surgery can effectively prevent the drying of organic contaminants and the formation of biofilms, thereby improving the quality of instrument cleaning (3). Timely preprocessing can remove contaminants from instrument surfaces and prevent instruments from coming into contact with oxygen and moisture in the air. This can slow oxidation, corrosion, and rusting on instrument surfaces, thereby extending the service life of the instruments (4). Therefore, standardized preprocessing of surgical instruments after use is a crucial step in ensuring surgical safety, preventing and controlling nosocomial infections, and reducing wear and tear on surgical instruments.

CSSD work standards and guidelines issued in China and other countries specify requirements for preprocessing surgical instruments after use. The Practice Specification for Pretreatment of Reusable Medical Devices released by the Chinese Research Hospital Association, the Standards for Operating Procedure of Cleaning, Disinfection and Sterilization released by the National Health and Family Planning Commission of China, and the Cleaning Technique for Medical Instruments released by the Chinese Nursing Association require instrument users to remove visible contaminants from instruments immediately after use; instruments for which cleaning cannot begin in the CSSD within one hour after use should be kept moist (2, 5, 6). ANSI/AAMI ST79:2017, Comprehensive Guide to Steam Sterilization and Sterility Assurance in Health Care Facilities, released by the Association for the Advancement of Medical Instrumentation and accredited by the American National Standards Institute, states that surgical instruments should be soaked or kept moist after use to prevent blood from drying and to avoid difficult decontamination (7). The AORN Guideline for Care and Cleaning of Surgical Instruments states that blood should be removed from instrument surfaces immediately after use, and that the instruments should remain moist before cleaning (8). Despite existing standards and guidelines, gaps persist in the quality of their implementation. These gaps present a major challenge to the prevention and control of nosocomial infections and to quality management in the CSSD. Previous studies have shown that, although surgical instrument preprocessing is generally well implemented, problems in procedures and methods remain (9, 10).

In practice, preprocessing personnel usually rely on their work experience when preprocessing surgical instruments (11–13). Although many effective preprocessing methods have been developed through practical experience and exploratory studies, research on systematic, standardized preprocessing remain limited. This evidence-based quality improvement project was conducted to address problems with the implementation of standards and guidelines, management challenges, and gaps in existing research. The study was reported in accordance with the Standards for Quality Improvement Reporting Excellence (SQUIRE) 2.0. The project involved a literature search, an assessment of literature quality, evidence extraction and grading, and an analysis of barriers to applying evidence in CSSD practice. Through this process, an evidence-based surgical instrument preprocessing protocol was developed that was adapted to and feasible in clinical settings. The evidence was then applied in practice, and its effects were evaluated. This quality improvement intervention aimed to standardize surgical instrument preprocessing procedures, strengthen staff awareness of evidence-based quality control, improve the pass rate for reusable surgical instrument cleaning, reduce the proportion of instruments returned to the decontamination area for initial re-cleaning, decrease the risk of hospital-acquired infections related to surgical instruments, and ensure patient safety.

## 2 Materials and methods

### 2.1 Study object

The evidence was applied in the CSSD of a tertiary Grade A hospital in Sichuan, China. The CSSD covers an area of 2,150m^2^, with a cleaning workload of approximately 11 million surgical instruments per year, and can simultaneously meet the sterile supply needs of about 2,000 beds. The CSSD had 20 nurses and 32 technical workers. This quality improvement project was carried out within the CSSD. The participants were CSSD staff members involved in surgical instrument preprocessing.

### 2.2 Study design

This study followed the Joanna Briggs Institute (JBI) model of evidence-based healthcare and adopted a four-step approach—evidence acquisition, baseline audit, evidence-based clinical application, and effect evaluation—to implement the evidence-based practice. This single-center quality improvement study used a one-group pretest-posttest design without a separate control or comparison group. Data collected before evidence implementation served as baseline data. Outcome measured before and after evidence implementation in the same clinical setting were compared to evaluate the effects of the standardized surgical instrument preprocessing protocol.

#### 2.2.1 Evidence summarization

##### 2.2.1.1 Creating an evidence-based practice panel

The evidence-based practice panel consisted of ten members. Among them, one was a head nurse engaging in cross-departmental nursing involving the CSSD, operating room, Anesthesiology Department, and Emergency Department and was responsible for the overall planning and technical guidance of the evidence-based practice; two were head nurses responsible for promoting practice implementation, coordinating personnel, and conducting quality control; two were research nurses who had received training in evidence-based nursing and were responsible for evidence retrieval and summarization, evidence appraisal and analysis, implementation of the audit criteria, and formulation of the implementation plan; and five were nurses who implemented the practice and collected and organized the data.

##### 2.2.1.2 Formulating the evidence-based questions

The evidence-based questions were formulated using the PIPOST (population, intervention, professional, outcome, setting, and type of evidence) framework. The scope of the questions were defined as follows: P—surgical instruments; I—preprocessing; P—surgical team members and CSSD staff; O—pass rate of surgical instrument cleaning; S—operating room and CSSD; T—guidelines, evidence summaries, systematic reviews, expert consensus statements, and primary research for clinical practice (14).

##### 2.2.1.3 Acquiring the best evidence

The following resources were searched to identify all relevant evidence based on the 6S Evidence Pyramid model (15): ① computerized decision support systems, including BMJ Best Practice and UpToDate; ② websites for accessing guidelines, including the Scottish Intercollegiate Guidelines Network (SIGN), Guidelines International Network (GIN), the United Kingdom’s National Institute for Health and Care Excellence (NICE), the United States Agency for Healthcare Research and Quality (AHRQ), the United States National Guideline Clearinghouse (NGC), the United States Association of periOperative Registered Nurses (AORN), Canada’s Registered Nurses’ Association of Ontario (RNAO), New Zealand Guidelines Group (NZGG), and China’s Yimaitong databases; ③ evidence-based databases, including the Cochrane Library and the JBI database; ④ primary research databases, including PubMed, Embase, Web of Science, China National Knowledge Infrastructure (CNKI), Wanfang Data, the China Science and Technology Journal Database, and the China Biology Medicine disc; and ⑤ healthcare-related websites, including the Chinese Nursing Association, the United States Association for the Advancement of Medical Instrumentation, the U. S. Food and Drug Administration, the World Gastroenterology Organization, and the European Society of Gastrointestinal Endoscopy. Gray literature was searched using Google Scholar.

The literature search was conducted from inception to January 29, 2025. A total of 26 studies were included. Of these, 15 were guidelines, 10 were expert consensus statements, and one was an evidence summary (Supplementary file 1). A total of 26 pieces of evidence were obtained from the included literature.

##### 2.2.1.4 Determining the audit criteria

The evidence-based practice panel evaluated the 26 pieces of evidence using the FAME (feasibility, appropriateness, meaningfulness, and effectiveness) strategy (16), based on workplace conditions, departmental resources, and their professional judgment. A total of 22 pieces of evidence were ultimately included in the study, and 20 audit criteria were derived from the evidence. Details of the evidence content, audit criteria, and audit methods are presented in Table 1. Compliance with the audit criteria was measured using the corresponding audit methods described in Table 1. “Yes (Y)” indicated compliance with an audit criterion, “no (N)” indicated noncompliance with an audit criterion, and “not applicable (NA)” indicated that the criterion was not measured. The compliance rate for each audit criterion was calculated as the percentage of Y responses out of the total number of measurements for that criterion.

**Table 1:** Evidence content, audit criteria and audit methods.

Category	Evidence content	Audit criteria	Audit methods
Point-of-use instrument preprocessing	After use, surgical instrument users immediately remove visible contaminants from instrument surfaces at the point of use.	After surgery, nurses or workers remove visible bloodstains, dirt and human tissues from the surfaces of surgical instruments.	On-site observations and interviews with the surgeons and operating room nurses.
	Enzyme-based preprocessing agents, including gels, foams, and sprays, are used to moisten the instruments. Appropriate enzyme-based preprocessing agents are selected based on compatibility with instruments and according to the instructions for use; alternatively, a wet cloth is placed over instruments to keep them moist until cleaning begins.	Enzyme-based preprocessing agents, including gels, foams, and sprays, or a wet cloth are used to keep instruments moist.	On-site observations.
	Preprocessing personnel must not use saline solutions, aldehydes, peracetic acid, or other products that are corrosive to instruments, cause rusting, or lead to protein coagulation for preprocessing of surgical instruments.		
	Oral instruments that have complex structures and are difficult to clean, such as dental handpieces and curettes, are kept moist and placed in collection containers, using softened water or enzyme-based preprocessing agents as the moistening agent.		
	The endoscope surface is immediately wiped with lint-free wet gauze at the end of each surgery while the endoscope remains connected to the light source.	After each use, the flexible endoscope surface is wiped with a lint-free cloth while the endoscope remains connected to the light source.	On-site observations and interviews with the surgeon and endoscopy nurse.
	For endoscopes with lumens, the distal ends are immersed in a container filled with cleaning solution, and the lumens are continuously flushed using 200–250 mL of liquid or for a duration of 10–20 s until no visible contaminants are seen in the fluid flowing from the lumens; proper cleaning solution is used according to the manufacturer’s instructions for use; visual inspections are performed on the endoscopes to identify any damage.	The insertion portion of the flexible endoscope is immersed into a container containing a cleaning agent; the lumen is continuously flushed until no visible contaminants are seen in the fluid flowing from the lumen; visual inspections are performed on the surface of the flexible endoscope to identify any indentations or damage.	On-site observations and interviews with the surgeon and endoscopy nurse.
	After the endoscope is disconnected from the light source, a water-resistant cap is attached to the connector of the endoscope, and the endoscope cable is wiped to remove contaminants from its surface.	The light source connector is removed from the flexible endoscope, a water-resistant cap is attached to the connector, and the endoscope cable surface is wiped.	On-site observations and interviews with the surgeon and endoscopy nurse.
	Lumened instruments should be disassembled as completely as possible after use. The inner cores are removed, and the lumens are flushed. Aerosol generation should be avoided.	After use, rigid endoscopes with lumens are disassembled according to their structure. The inner cores are removed, and the lumens are flushed.	On-site observations and assessment
	Dental handpieces, electric scalers and electrosurgical units are stored in a dry collection container after preprocessing.	Dental handpieces, electric scalers and electrosurgical units are stored in dry collection containers.	On-site observations.
	After use of viscoelastic agent, instruments are immediately immersed in sterile water.	Ophthalmic instruments are immersed in sterile water immediately after viscoelastic agents are used.	On-site observations and interviews with surgeons and operating room nurses.
	Distilled water or deionized water (with a bacterial concentration <10 cfu/mL), or other liquids specified in the instrument manufacturer’s instructions for use, are used for preprocessing of intraocular surgical instruments; enzyme cleaners are not routinely recommended.	Intraocular surgical instruments are rinsed, wiped, and preprocessed using deionized water (with a bacterial concentration <10 cfu/mL).	On-site observations.
	Ophthalmic instruments are separated from non-ophthalmic instruments throughout the entire preprocessing procedure and must not be mixed with other instruments during loading.	Ophthalmic instruments are placed in their dedicated collection containers.	On-site observations.
	Reusable and disposable instruments are placed separately at the point of use. The disposable instruments are disposed properly after use.	Disposable instruments, such as blades and sutures, are discarded at the point of use.	On-site observations.
Instrument transfer	After preprocessing is completed at the point of use, contaminated instruments are promptly transported to the CSSD decontamination area, with proper anti-collision protection measures for delicate surgical instruments during transport. This can help improve the efficiency and effectiveness of instrument cleaning.	Surgical instruments are transported to the CSSD decontamination area within 1 hour after preprocessing. Delicate surgical instruments must be placed in containers with fixture slots or silicone mats for transportation. Flexible endoscopes are placed in dedicated collection containers, while rigid endoscopes are placed in containers with fixture slots.	On-site observations.
	Endoscopes and laparoscopes are promptly transported to the CSSD for reprocessing after preprocessing at the point of use, ideally within 15 min, but no later than 1 hour. Endoscopes must be placed in separate containers and transported apart from other instruments to prevent damage.		On-site observations.
	Instruments should be placed in sealed containers, such as a cabinet with a lid, during transportation, clearly labeled with the type of contamination. The external surfaces of the transport containers remain uncontaminated to prevent environmental pollution.	Surgical instruments are placed in sealed containers, such as a cabinet with a lid, during transportation, clearly labeled with the type of contamination. The external surfaces of the transport containers remain uncontaminated.	On-site observations.
Preprocessing at the CSSD decontamination area	CSSD staff sort instruments at the decontamination area according to their degrees of contamination, precision and structures; follow the instructions for use to select appropriate methods and cleaning agents for soaking and manual cleaning; and disassemble contaminated instruments into the smallest possible units.	CSSD staff sort instruments at the decontamination area according to their degree of contamination, precision, and structures, and then disassemble the instruments. Severely contaminated instruments are soaked and scrubbed before cleaning.	On-site observations and assessment.
	CSSD staff should remove all valves and buttons before cleaning, perform a leak test on the instruments according to the manufacturer’s instructions for use, and inspect the integrity of all channels.	CSSD staff remove all accessories from the flexible endoscopes prior to cleaning, inspect endoscope surfaces for indentations and damage, and perform a leak test.	On-site observations and assessment.
	Cleaning tools for preprocessing, such as brushes and cloths, are disposable and are discarded after use.	All cleaning tools for preprocessing are for single use only.	On-site observations.
	Cleaning agents or water used for lumen flushing must be disposable and discarded after use.	Cleaning agents or water used for lumen flushing are for single use only.	On-site observations.
Staff training and management	Surgical instruments are preprocessed by designated personnel who have received professional training and possess the necessary competence.	All preprocessing personnel have received relevant training and passed assessments of preprocessing knowledge and skills.	On-site observations and assessment.
	Operating room and related departments should provide ongoing education and training to instrument preprocessing personnel, including instructions for use from instrument manufacturers, preprocessing procedures, instrument transport, and personal protective equipment, and should conduct regular assessments.	Four preprocessing training sessions are conducted annually, each followed by an assessment and documentation. Routine quality control includes random checks of preprocessing knowledge and assessments of practical skills.	On-site observations.

In this study, most criteria relate to preprocessing procedures were audited 60 times before and 60 times after the evidence was applied in practice. Observations were distributed evenly across different days, morning and afternoon shifts, and all stages of instrument preprocessing. Observations were conducted at predefined intervals rather than consecutively. Simple random sampling was not used. Because staff rotated within the CSSD, the same staff member could be observed multiple times. However, each observation corresponded to a separate operational event, and no individual operation was observed more than once. This approach was intended to ensure the independence of the observations. Criteria 19 and 20 involved staff training and management and were audited once before and once after the application of evidence. “1” indicated the presence of relevant training or records, while “0” indicated the absence of relevant training or records. The standardized audit checklist for the 20 audit criteria is presented in Supplementary file 2 and can be used for subsequent research. Compliance with the audit criteria was evaluated through on-site observations. CSSD staff members were not told when observations would take place, to minimize the Hawthorne effect. All CSSD staff members knew that the project involved routine quality control monitoring; the nature of the project itself was not concealed. The observation plan was included in the ethics application, and the requirement for written informed consent was waived. No personally identifiable information about staff members was recorded during observations. Only information about compliance with the audit criteria for preprocessing procedures were collected for the overall quality evaluation.

Before the baseline survey was initiated, this study used the same database, search strategy, and inclusion and exclusion criteria as the initial search to conduct a supplementary search for literature published from February 1 to October 1, 2025, to retrieve new guidelines, expert consensus statements, and relevant evidence. No new evidence meeting the inclusion criteria was found, so no additional literature was included. This study checked the publication dates of all included evidence to ensure that they were consistent with the original search records.

#### 2.2.2 Baseline audit before the application of evidence

The baseline audit period was from October 1 to 30, 2025. The evidence-based practice panel conducted the baseline audit and calculated criterion compliance rates using the methods described above. Meanwhile, an assessment of surgical instrument preprocessing knowledge was conducted among 52 CSSD staff members. The assessment form (Supplementary file 3) was designed by the evidence-based practice panel. It contained 10 questions, and respondents selected answers from the given response options. Respondents received a score of 10 for each question answered correctly. The maximum total score was 100. The assessment was conducted using Wenjuanxing, a popular online survey and assessment platform in China. The assessment content was developed based on the evidence included in this study and CSSD guidelines. It was reviewed and revised by two senior CSSD nursing experts. It was used to assess CSSD staff’s mastery of instrument preprocessing knowledge. Because it was a knowledge assessment, it did not undergo psychometric evaluation of reliability and validity. The same 52 CSSD staff members completed the knowledge assessment before and after the evidence was applied. Each staff member completed both assessments, providing complete paired data with no missing values. Normality tests were performed on the differences between paired knowledge assessment scores before and after evidence implementation. These differences followed a normal distribution; therefore, a paired-samples *t*-test was used for the within-group comparison.

#### 2.2.3 Applying the evidence

The evidence-based practice panel discussed the barriers to applying evidence to surgical instrument preprocessing based on the baseline audit results. They analyzed the causes of these barriers and formulated solutions tailored to the CSSD setting to apply the best evidence in surgical instrument preprocessing. The identified barriers and countermeasures were as follows:

(1) *Barrier 1*. The baseline audit results revealed poor surgical instrument preprocessing and low compliance with the criteria. The barriers included the lack of a dedicated work area for surgical instrument preprocessing, the absence of designated preprocessing personnel because of rotations between preprocessing and other positions, and an unclear division of preprocessing responsibilities. The countermeasures taken were as follows: ① a dedicated work area for instrument preprocessing was established in the operating room to achieve physical separation from other functional areas; ② designated CSSD personnel were assigned to the operating room to be responsible for preprocessing contaminated surgical instruments, as well as for instrument collection and transportation to the CSSD. They were the personnel primarily accountable for the quality of surgical instrument preprocessing, including traceability management of surgical instruments and effective communication with operating room staff in the event of instrument defects or errors; ③ a WeChat group was created for the clinical departments and the CSSD. When failures in surgical instrument preprocessing were identified, staff could immediately photograph the issue and send it to the group chat for feedback and to remind the relevant personnel to address it.
(2) *Barrier 2*. The baseline audit results showed that personnel responsible for surgical instrument preprocessing lacked sufficient knowledge of preprocessing. The CSSD provided insufficient ongoing training on preprocessing and lacked a regular assessment system. The barriers included an inadequate preprocessing training system and the absence of mandatory pre-onboarding training and regular post-training assessment mechanisms. The countermeasures taken were as follows: ① a standardized training management system that included mandatory pre-onboarding and regular in-service training was developed. It clearly stated that pre-onbarding and in-service training were mandatory for preprocessing personnel; ② a standardized training and assessment system was developed. It included instrument manufacturers’ instructions for use, preprocessing procedures, instrument transportation, and protective measures for preprocessing personnel; ③ CSSD personnel were permitted to work at the preprocessing position only after passing the pre-onboarding training assessment. Regular theoretical and practical skill assessments were conducted, and eligibility to work at the instrument preprocessing position and work performance evaluations were directly linked to post-training assessment results.
(3) *Barrier 3*. The baseline audit results revealed an insufficient supply of enzyme-based preprocessing agents, a lack of dedicated collection containers for special medical devices (such as dental and ophthalmic devices), inadequate anti-collision protection for delicate instruments, and the reuse of special-purpose water (such as distilled, deionized, and sterile water) and disposable cleaning products due to delayed supply. The barrier was an inadequate preprocessing material supply system. The countermeasures taken were as follows: ① enzyme-based preprocessing agents were maintained at the maximum stock level, demand was calculated based on daily workload, and inventory management was improved. An inventory alert system was established, and a designated person was assigned to record inventory counts to ensure a regular, continuous supply; ② dedicated collection containers were provided for special medical devices, such as dental and ophthalmic devices. The containers were clearly labeled to ensure sorted collection and separate transportation. This helped avoid mixing different devices and reduced the risk of damage; ③ sufficient anti-collision protection supplies were provided for delicate instruments. The anti-collision requirements for each step were clarified to standardize the operating process and reduce the risk of instrument damage; ④ sufficient special-purpose water and disposable cleaning products were supplied. An inventory alert system was established, and the inventory management system was strictly implemented. The reuse of disposable products was prohibited.
(4) *Barrier 4*. The baseline audit results revealed that some surgical instruments were inadequately preprocessed. For example, surgical instruments were not disassembled to the smallest units before cleaning, lumens were not flushed, and contaminated sharp instruments and used disposable items (such as blades and cotton swabs) were placed together with other surgical instruments. The barriers included inadequate preprocessing procedures for different types of instruments and an inadequate CSSD supervision system. The countermeasures taken were as follows: ① standard operating procedures for preprocessing different types of instruments were formulated, and relevant training and assessment were provided for preprocessing personnel; ② assessment criteria for preprocessing different instruments were developed to standardize preprocessing quality management; ③ a three-level quality management system for surgical instrument preprocessing was established, with preprocessing nurses at Level 1, the head of the decontamination area at Level 2, and the head nurse at Level 3. The head of the decontamination area and the head nurse used a preprocessing inspection form to conduct daily quality control with the preprocessing personnel assigned to the operating room and the CSSD cleaning personnel to assess preprocessing procedures and the quality of instrument cleaning. Inspection results were shared in the WeChat group chat between the clinical departments and the CSSD; ④ preprocessing personnel whose preprocessing was inadequate were given individualized guidance. Inspection results were incorporated into work performance assessments to increase criterion compliance rates.

#### 2.2.4 Audit after the application of evidence

The evidence application lasted for 2 months. An audit was conducted from January 1 to 30, 2026, after the evidence was applied, using the same audit methods as those used in the baseline audit.

### 2.3 Data collection

Two CSSD nurses served as quality controllers, conducting on-site observations and collecting data. Before data collection, they received standardized training to clarify the definitions of the audit criteria and the requirements for a “pass” and to improve their understanding of these requirements through case studies. Regular joint calibration sessions were held during data collection. In ambiguous operational situations or those that were difficult to assess, the two quality controllers discussed their findings and reached a consensus before recording the results. Inter-observer agreement was not assessed quantitatively, leaving a risk of measurement variation. An audit report form for surgical instrument preprocessing was developed based on the 20 audit criteria. The quality controllers conducted on-site observations and recorded audit results to compare the compliance rates for each criterion before and after evidence application.

### 2.4 Statistical analysis

SPSS 25.0 was used for data analysis. Criterion compliance rates and the rate of instruments returned to the decontamination area for initial re-cleaning were described using counts and percentages. For comparisons between groups, Pearson’s chi-square test was used when expected cell counts were ≥5, and a two-tailed Fisher’s exact test was used when expected cell counts were < 5. The risk ratio and absolute risk difference, with 95% confidence intervals (CIs), were calculated for the proportion of surgical instruments returned to the decontamination area for initial re-cleaning. Total knowledge assessment scores were summarized as the mean ± standard deviation. A paired-samples *t*-test was used to compare scores before and after evidence implementation. The significance level (α) was set at 0.05. A statistically significant difference was defined as *p* < 0.05.

### 2.5 Ethical approval

Ethical approval of this study was obtained from the Medical Research Ethics Committee of West China Second University Hospital, Sichuan University on January 20, 2025 [2024 Medical Scientific Research for Ethical Approval No. (441)]. The ethics committee reviewed the research proposal, including the knowledge assessment plan, the concealed on-site observation plan, and the standardized script for obtaining verbal informed consent. Because the study posed minimal risk and the research proposal was feasible, the committee approved verbal informed consent without requiring participants to sign a written consent document. During the study, researchers verbally informed participants in accordance with the approved script and recorded the verbal informed consent process for every participant.

## 3 Results

### 3.1 Criterion compliance rates before and after the application of evidence

Among the 20 audit criteria, the compliance rates for all except criteria 5, 14 and 16, increased significantly after the application of evidence (*p* < 0.05). Criteria 19 and 20 concerned staff training and management and were audited once before and once after the application of evidence. Training on surgical instrument preprocesing was not provided before the application of evidence but was provided after the application of evidence. Because of the extremely small sample size, only descriptive analysis was performed without hypothesis testing (Table 2).

**Table 2:** Criterion compliance rates before and after the application of evidence.

Audit criteria	Before the application of evidence (*n* = 60)			After the application of evidence (*n* = 60)			χ^2^	*p*-value
	Compliance	Noncompliance	Compliance rates (%)	Compliance	Noncompliance	Compliance rate (%)		
Criterion 1	15	45	25.00%	56	4	93.33%	57.982	<0.001
Criterion 2	25	35	41.67%	58	2	96.67%	42.553	<0.001
Criterion 3	54	6	90.00%	60	0	100.00%	NA	0.027
Criterion 4	52	8	86.67%	60	0	100.00%	NA	0.006
Criterion 5	57	3	95.00%	60	0	100.00%	NA	0.244
Criterion 6	19	41	31.67%	59	1	98.33%	58.608	<0.001
Criterion 7	7	53	11.67%	60	0	100.00%	94.925	<0.001
Criterion 8	28	32	46.67%	60	0	100.00%	43.636	<0.001
Criterion 9	8	52	13.33%	57	3	95.00%	80.593	<0.001
Criterion 10	10	50	16.67%	58	2	96.67%	78.190	<0.001
Criterion 11	42	18	70.00%	59	1	98.33%	18.072	<0.001
Criterion 12	30	30	50.00%	58	2	96.67%	33.409	<0.001
Criterion 13	48	12	80.00%	59	1	98.33%	10.439	0.001
Criterion 14	57	3	95.00%	60	0	100.00%	NA	0.244
Criterion 15	43	17	71.67%	60	0	100.00%	19.806	<0.001
Criterion 16	55	5	91.67%	60	0	100.00%	NA	0.057
Criterion 17	11	49	18.33%	52	8	86.67%	56.174	<0.001
Criterion 18	14	46	23.33%	58	2	96.67%	67.222	<0.001
Criterion 19	0	1	0	1	0	100%	–	–
Criterion 20	0	1	0	1	0	100%	–	–

A two-tailed Fisher’s exact test was performed on criteria with cell expected counts <5 or total effective evaluable sample size (*n*) < 5, while Pearson’s chi-square test was performed for other criteria. NA in the column for χ^2^ value indicated that Fisher’s exact test was performed. “–” meant that descriptive analysis was performed on criteria 19 and 20 because of insufficient statistical power caused by the small sample size.

### 3.2 CSSD staff’s total scores for the knowledge assessment before and after the application of evidence

CSSD staff’s mean total score for the knowledge assessment before the application of evidence was 58.06 ± 9.097, and it increased to 95.83 ± 3.930 after the application of evidence, with a difference of 37.78 (95% CI: 34.24–41.32, *t* = 22.520, *p* < 0.001).

### 3.3 Rates of instruments returned to the decontamination area for initial re-cleaning before and after the application of evidence

Among 615,868 surgical instruments cleaned during the baseline audit before the application of evidence, 4,148 returned to the decontamination area for re-cleaning, yielding a re-cleaning rate of 0.67%. Among 790,891 surgical instruments cleaned during the audit after the application of evidence, 672 returned to the decontamination area for re-cleaning, yielding a re-cleaning rate of 0.085%. Elective surgeries were suspended during China’s one-week National Day holiday during the baseline audit period, leading to a difference in the number of surgical instruments cleaned during the audit period and after the application of evidence. The re-cleaning rate differed significantly before and after the application of evidence (χ^2^ = 3512.527; degree of freedom = 1; *p* < 0.001; absolute risk difference = 0.58%, with a 95% CI of 0.56–0.60%; risk ratio = 7.93, with a 95% CI of 7.31–8.60).

## 4 Discussion

### 4.1 Implementing a closed-loop management system by dispatching CSSD staff to the surgical instrument preprocessing position in the operating room to enhance criterion compliance rates

The evidence-based surgical instrument preprocessing management scheme developed in this study effectively increased the criterion compliance rates of preprocessing personnel. During the baseline audit, the average compliance rate for the 20 audit criteria was low because of several weaknesses in preprocessing at that time, including the absence of a dedicated instrument preprocessing area in the operating room, the absence of designated preprocessing personnel due to rotations between preprocessing and other positions, unclear job responsibilities, and insufficient inventory. This is consistent with the findings of Wan et al. and Huang et al. (9, 10). In response to these barriers, our study implemented a refined dispatching model in which CSSD personnel were assigned to the instrument preprocessing position in the operating room. This was not a simple personnel assignment; rather, it involved formulating detailed implementation rules for the process. Additionally, multiple measures, such as sufficient inventory supply, real-time feedback, and a three-level quality control system, were implemented to form a closed-loop management system, thereby standardizing the practices of preprocessing personnel and ensuring the implementation of evidence-based practice. This closely aligns with the results of Chen et al. (17), who found that dispatching CSSD personnel to the instrument preprocessing position in the operating room could significantly improve the quality of preprocessing. In our study, the compliance rates for audit criteria 1 and 6 before the application of evidence were 25.00 and 31.67%, respectively, and they increased to 93.33 and 98.33%, respectively, after the application of evidence. The compliance rates for audit criteria 7 and 9 before the application of evidence were both below 15%, and they rose to 100.00 and 95.00% respectively, after the application of evidence. These findings demonstrate the positive role of evidence-based practice in instrument preprocessing.

In this study, the compliance rates for some audit criteria did not differ significantly because their baseline values were relatively high, at over 90%, indicating limited room for improvement. Among these, the compliance rate for Criterion 16 before the application of evidence was 91.67%, with a *p* value of 0.057, which was slightly above the significance level. This suggests that the implementation of this criterion still needs to be strengthened. Because Criterion 16 involves endoscope preprocessing, it is necessary to provide CSSD personnel dispatched to the preprocessing position in the operating room with training in endoscope leakage testing. This could refine the operating procedures for Criterion 16 and promote its continuous optimization.

### 4.2 Standardizing preprocessing procedures and quality control management to improve the quality of surgical instrument cleaning

In this study, the rate of instruments returned to the decontamination area for initial re-cleaning decreased from 0.67% before the application of evidence to 0.085% after the application of evidence, with a statistically significant difference (χ^2^ = 3512.527, *p* < 0.001). This directly reflects an improvement in the quality of surgical instrument preprocessing under evidence-based practice. It also reflects the core practical effect of implementing evidence-based measures. The high re-cleaning rate of 0.67% identified during the baseline audit was due to nonstandardized preprocessing, such as lumened instruments not being disassembled into the smallest unit before cleaning, lumens being flushed without measures to prevent aerosol generation, contaminants on instrument surfaces drying because instruments were not kept moist immediately after use, special devices (such as oral and ophthalmic devices) not being preprocessed according to special procedures, and sharp instruments or disposable items being placed together with other surgical instruments after use. These issues led to incomplete cleaning of the instruments, thereby increasing the rate of instruments returned to the decontamination area for re-cleaning.

After the application of evidence, designated CSSD personnel were assigned to the preprocessing position in the operating room, enabling professional preprocessing. A three-level quality management system was established, comprising nurses at the preprocessing position, the head of the decontamination area, and the head nurse. The head of the decontamination area and the head nurse conducted daily quality control using inspection forms and provided individualized guidance to preprocessing personnel whose practices were nonstandard. The inspection results were incorporated into work performance evaluations. Standardized preprocessing procedures for different types of instruments, such as lumened, oral, and ophthalmic devices, were formulated. The significant reduction in the rate of instruments returned to the decontamination area for initial re-cleaning effectively reduced repetitive work in the CSSD and improved the efficiency of instrument preprocessing. It also reduced the risk of damage to delicate instruments caused by repeated cleaning and handling, thereby extending their service life (18). More importantly, improvements in instrument preprocessing quality provided a solid foundation for subsequent disinfection and sterilization, ensuring the safe use of instruments in clinical practice. This reflects the value of both clinical quality control and operational management.

### 4.3 Building a standardized training and assessment system to improve CSSD staff’s knowledge of instrument preprocessing

Evidence-based practice emphasizes that clinical skills are essential to effective evidence implementation and that staff members’ professional knowledge forms the foundation of these skills (19). In this study, a standardized training and assessment system, including mandatory pre-onboarding training and regular assessments of theoretical knowledge and practical skills, was developed as part of the evidence-based practice initiative in response to an inadequate preprocessing training system, the lack of mandatory pre-onboarding training, and the absence of a regular training and assessment system. The mean score for CSSD staff’s knowledge of instrument preprocessing rose from 58.06 ± 9.097 before the application of evidence to 95.83 ± 3.930 after the application of evidence, with a statistically significant difference (*t* = 22.520, *p* < 0.001). This indicated that CSSD staff had significantly improved their knowledge of instrument preprocessing after the application of evidence. The improvement in staff knowledge provided a solid foundation for dispatching CSSD staff to the preprocessing position in the operating room and for standardizing preprocessing procedures. This is consistent with the results of Ji et al. (20), who found that systematic training is essential for improving the professional knowledge and skills of personnel involved in instrument preprocessing. CSSD staff’s adequate understanding of the criteria and significance of preprocessing can improve their compliance with standardized operations, thereby promoting the effective translation of evidence into practice.

In this study, post-training assessment results were incorporated into evaluations of eligibility to work at the instrument preprocessing position and into work performance assessments, reflecting both incentive and accountability mechanisms and effectively addressing the persistent problems of insufficient training and lack of post-training assessment. This promoted the translation of knowledge into standardized behavior. In summary, developing a full-cycle training system that includes pre-onboarding requirements, in-service training, and regular assessment is an important institutional foundation for improving preprocessing personnel’s job competence and ensuring the quality of instrument preprocessing.

### 4.4 Limitations

This study has several limitations. First, it was a single-center, uncontrolled study with a one-group pretest-posttest design. Outcomes were compared with baseline data, and no separate control or comparison group was established. The effects of confounding factors, including changes over time, other hospital interventions to prevent nosocomial infections during the same period, and adjustments to management policies, could not be ruled out. The improvements observed cannot be directly attributed to the evidence implementation measures, and the findings should be extrapolated with caution. Second, sample sizes differed significantly across outcomes. The instrument re-cleaning rate was based on more than six hundred thousand instruments, whereas the knowledge assessment included only the same 52 staff members. Each audit criteria related to operational procedures, excluding those related to staff training or management, was assessed through only 60 on-site observations before and 60 after evidence implementation. This limited the ability to detect infrequent nonstandard practices. The single-center setting also limited the generalizability of the findings, which require validation in multicenter practice. The observation period following evidence implementation lasted only 2 months. Follow-up is needed to assess the long-term effects of assigning CSSD staff to instrument preprocessing duties in the operating room, their long-term compliance with the audit criteria, and the sustained effects of these criteria. Third, the study may be subject to bias. Although staff members were not told in advance when observations would take place, the Hawthorne effect could not be completely eliminated. Staff who realized they were being observed might have improved their compliance with preprocessing criteria, potentially leading to an overestimate of the improvement resulting from evidence implementation. Instrument re-cleaning data were retrieved from a hospital traceability system. The effectiveness of evidence implementation was assessed using both process criteria and objective quality criteria, helping to address the limitations of subjective observations. However, inter-observer agreement was not assessed quantitatively, leaving a potential risk of measurement variation. Fourth, observations might have been clustered and not fully independent. The instrument re-cleaning rate was calculated using individual instruments as the statistical unit, and the analysis included multiple instruments from the same surgery, instrument tray, or preprocessing batch. Each on-site observation concerned a single preprocessing event, but the same staff member could be observed multiple times, creating possible correlations between observations. The strict assumption of independence required by the chi-square test could therefore not be fully met. Because the research did not include a predefined multilevel statistical model, the chi-square test results were used to describe the magnitude of changes before and after evidence implementation. Statistical inferences should be interpreted cautiously in the presence of clustering effects. Fifth, elective surgeries were suspended during China’s one-week National Day holiday, which fell within the baseline audit period. This resulted in different numbers of surgical instruments being preprocessed during the baseline period and the period following evidence implementation. The hospital traceability system showed no differences in surgical specialties or instrument types between the two periods. However, re-cleaning data were summarized and lacked precise timestamps, preventing a sensitivity analysis that separated out the holiday data. The potential impact of the holiday could not be ruled out when interpreting changes in the re-cleaning rate.

## 5 Conclusion

This study integrated the best evidence for surgical instrument preprocessing into clinical practice. It addressed the core barriers through various countermeasures, such as dispatching CSSD staff to the preprocessing position in the operating room, improving the training system and quality control, and strengthening inventory management, thereby effectively reducing potential infection risks and supporting the optimization of nosocomial infection prevention and control system. This study can provide reliable evidence for improving the quality of surgical instrument preprocessing, thereby helping prevent and control nosocomial infections.

## Data Availability

The raw data supporting the conclusions of this article will be made available by the authors, without undue reservation.

## References

[1] Evangelista SS Guimaraes NR Garcia NB Santos SGD Oliveira AC. Effectiveness of manual versus automated cleaning on *Staphylococcus epidermidis* biofilm removal from the surface of surgical instruments. Am J Infect Control. (2020) 48:267–74. doi: 10.1016/j.ajic.2019.08.024, 31630921

[2] Chinese Research Hospital Association. T/CRHA 079-2024 Practice Specification for Pretreatment of Reusable Medical Devices. Beijing: Standards Press of China (2024).

[3] Weber DJ Rutala WA Anderson DJ Sickbert-Bennett EE. Biofilms on medical instruments and surfaces: do they interfere with instrument reprocessing and surface disinfection. Am J Infect Control. (2023) 51:A114–9. doi: 10.1016/j.ajic.2023.04.158, 37890940

[4] Cao XL Yan ZJ. Effect of pre-moisturising combined with partial descaling on the cleaning quality of old instruments. Chin J Disinfect. (2022) 39:749–52. doi: 10.11726/j.issn.1001-7658.2022.10.010

[5] National Health and Family Planning Commission of the People’s Republic of China. WS 310.2-2016 Central sterile Supply Department (CSSD)-Part 2: Standard for Operating Procedure of Cleaning, Disinfection and Sterilization. Beijing: Standards Press of China (2016).

[6] Chinese Nursing Association. T/CNAS 09-2019 Cleaning Technique for Medical Instruments. Beijing: Standards Press of China (2024).

[7] Association for the Advancement of Medical Instrumentation. ANSI/AAMI ST79: 2017 Comprehensive Guide to Steam Sterilization and Sterility Assurance in Health care Facilities. Arlington: Association for the Advancement of Medical Instrumentation (2017).

[8] Link T. Guidelines in practice: instrument cleaning. AORN J. (2021) 114:241–51. doi: 10.1002/aorn.13495, 34436771

[9] Wan XC Zhou SP Yu SW Xu CY Ma CJ Chen J. Investigation and analysis of surgical instrument pretreatment in medical institutions of Hainan province. Chin Nurs Manag. (2022) 22:290–3. doi: 10.3969/j.issn.1672-1756.2022.02.024

[10] Huang LL Zhu J Yin SY Ding XQ Chen S Wang Y. The current investigation status of on-site pretreatment on reusable medical devices in the hospital of Hubei Province. J Nurs Adm. (2024) 24:237–41. doi: 10.3969/j.issn.1671-315x.2024.03.011

[11] Li AQ Zhang HZ Sun XY Bai CM Wang XL Zhou RY. Effect of foam pretreatment humectant on cleaning quality of lumen instruments. Chin J Nosocomiol. (2022) 32:317–20. doi: 10.11816/cn.ni.2022-210514

[12] Jiang XM Song Y Fang F Qi J Shao W. A study on the impact of different pretreatment approaches on the cleaning quality of reusable medical devices. Chin J Disinfect. (2023) 40:473–5. doi: 10.11726/j.issn.1001-7658.2023.06.022

[13] Fu XY Jiang Y Zheng SH. A comparative study on the cleaning efficacy of surgical instruments with different pretreatment methods. Chin J Disinfect. (2022) 39:316–8. doi: 10.11726/j.issn.1001-7658.2022.04.023

[14] Ouyang J Gu Y Yuan Y Chen GL Xu JQ Tao FF . Evidence application for wet pack management. Chin Nurs Manag. (2021) 21:1452–6. doi: 10.3969/j.issn.1672-1756.2021.10.003

[15] Dicenso A Bayley L Haynes RB. Accessing pre-appraised evidence: fine-tuning the 5S model into a 6S model. Evid Based Nurs. (2009) 12:99.2–99.101. doi: 10.1136/ebn.12.4.99-b, 19779069

[16] Jordan Z Lockwood C Munn Z Aromataris E. Redeveloping the JBI model of evidence based healthcare. Int J Evid Based Healthc. (2018) 16:227–41. doi: 10.1097/XEB.0000000000000139, 29664758

[17] Chen YZ Fu LM Chen YB Lin XZ. A study on the application of forward deployment management of the central sterile supply department in the preprocessing of surgical instruments. Chin J Disinfect. (2023) 40:706–8. doi: 10.11726/j.issn.1001-7658.2023.09.020

[18] Gao XZ Yang J Lin SH Li ZY Zhang YY Zhang YL. Practical exploration of precision surgical instrument project management in disinfection supply center. J Nurs Sci. (2023) 38:75–7. doi: 10.3870/j.issn.1001-4152.2023.10.075

[19] Zhang KL Li YQ Li YS Chen J Huang HN Yang LF. The best evidence application for cleaning metal tube instruments with suction head. Chin J Disinfect. (2025) 42:292–6. doi: 10.11726/j.issn.1001-7658.2025.04.017

[20] Ji XQ Wang LJ Qi WD. Effect of team-based learning on nursing teaching in disinfection supply center. Chin J Disinfect. (2022) 39:602–5. doi: 10.11726/j.issn.1001-7658.2022.08.014

