# Supplementary material for: Evidence-based preprocessing improvement bundle for surgical instruments: reducing surgical infection risks via quality improvement
Source: Front Public Health. 2026 Oct 27;14:1930642. doi: 10.3389/fpubh.2026.1930642 (PMC13761332; doi:10.3389/fpubh.2026.1930642)
Supplement: Supplementary file 1 [file Supplementary_file_1.docx]

**Supplementary file 1** Characteristics of included studies

| Included studies | Topic | Literature sources | Literature type | Year of publication/update |
| --- | --- | --- | --- | --- |
| Association for the Advancement of Medical Instrumentation^[1]^ | Comprehensive guide to steam sterilization and sterility assurance in health care facilities | NGC | Guidelines | 2017 |
| Asia Pacific Society of Infection Control^[2]^ | APSIC guidelines for disinfection and sterilization of instruments in health carefacilities | Pubmed | Guidelines | 2018 |
| Association for perioperative Registered Nurses^[3]^ | Guidelines in practice: Instrument cleaning | AORN | Guidelines | 2021 |
| National Health and Family Planning Commission of the People’s Republic of China WS 310^[4]^ | Standard for operating procedure of cleaning, disinfection and sterilization | CNKI | Expert Consensus | 2016 |
| The Society for Healthcare Epidemiology of America^[5]^ | Guideline for disinfection and sterilization of prioncontaminated medical instruments | Pubmed | Guidelines | 2010 |
| The National Health Service in England^[6]^ | Health Technical Memorandum 01 -01: Decontamination of reusable medical devices | SIGN | Guidelines | 2017 |
| Chinese Nursing Association^[7]^ | Cleaning technique for medical instruments | Chinese Nursing Association | Expert Consensus | 2019 |
| Centers for Disease Control and Prevention^[8]^ | Guideline for disinfection and sterilization in healthcare facilities | CDC | Guidelines | 2019 |
| Kommission für Krankenhaushygiene und Infektionsprävention, et al.^[9]^ | Hygiene requirements for thereprocessing of medical devices | Pubmed | Expert Consensus | 2012 |
| American Society for Gastrointestinal Endoscopy^[10]^ | ASGE guideline for infection control during GI endoscopy | Pubmed | Guidelines | 2018 |
| Gastroenterological Society of Australia^[11]^ | Infection prevention and control in endoscopy | GESA | Guidelines | 2021 |
| World Gastroenterology Organisation^[12]^ | Endoscope disinfection update:A guide to resource sensitive reprocessing | WGO | Guidelines | 2019 |
| European Society of Gastrointestinal Endoscopy and European Society of Gastroenterology Nurses and Associates^[13]^ | Reprocessing of flexible endoscopes and endoscopic accessories used in gastrointestinal endoscopy | Pubmed | Expert Consensus | 2018 |
| Meyer GW, et al.^[14]^ | Preventing infection transmitted by gastrointestinal endoscopy | Up To Date | Evidence Summary | 2022 |
| National Health and Family Planning Commission of the People’s Republic of China WS 507^[15]^ | Regulation for cleaning and disinfection technique of flexible endoscope | CNKI | Expert Consensus | 2016 |
| The French Agency for Safety of Health Products^[16]^ | Importance of cleaning for reprocessing endoscopes and thermolabile sterile medical devices | Pubmed | Expert Consensus | 2004 |
| The National Health Service in England^[17]^ | Health Technical Memorandum 01-06 :Decontamination of flexible endoscopes | SIGN | Guidelines | 2016 |
| The Society of Gastroenterology Nurses and Associates^[18]^ | Standards of infection prevention in reprocessing flexible gastrointestinal endoscopes | Pubmed | Guidelines | 2020 |
| Asia Pacific Society of Infection Control^[19]^ | APSIC dental infection prevention and control (IPC) guidelines | Pubmed | Guidelines | 2023 |
| National Health and Family Planning Commission of the People’s Republic of China WS 506^[20]^ | Regulation for disinfection and sterilization technique of dental instruments | CNKI | Expert Consensus | 2016 |
| The National Health Service in England^[21]^ | Health Technical Memorandum 01-05:Decontamination in primary care dental practices | SIGN | Guidelines | 2013 |
| The Ophthalmic Instrument Cleaning and Sterilization Task Force^[22]^ | Guidelines for the cleaning andsterilization of intraocular surgical instruments | Pubmed | Guidelines | 2018 |
| Ophthalmologist Branch of the Chinese Medical Doctor Association, et al^[23]^ | Guidelines for Ophthalmic Surgery Management, Infection Control, and Disinfection and Sterilization in China | Wanfang | Guidelines | 2016 |
| Ophthalmic Nursing Committee of the Chinese Nursing Association, et al^[24]^ | Expert Consensus on Cleaning Methods forOphthalmic Surgical Instruments Attached toSilicone Oil | Wanfang | Expert Consensus | 2020 |
| Chinese Research Hospital Association^[25]^ | Practice specification for pretreatment of reusable medical devices | Wanfang | Expert Consensus | 2024 |
| Cleaning and Disinfection Group of the Digestive Endoscopy Branch of the Chinese Medical Association^[26]^ | Interpretation of Chinese expert consensus on gastrointestinal endoscope reprocessing | Wanfang | Expert Consensus | 2024 |

^[1]^Association for the Advancement of Medical Instrumentation. ANSI/AAMI ST79:2017 Comprehensive guide to steam sterilization and sterility assurance in health care facilities. Arlington:Association for the Advancement of Medical Instrumentation. 2017.

^[2]^Ling ML, Ching P, Widitaputra A, Stewart A, Sirijindadirat N, Thu LTA. APSIC guidelines for disinfection and sterilization of instruments in health care facilities. Antimicrob Resist Infect Control. 2018;7:25. doi: 10.1186/s13756-018-0308-2.

^[3]^Link T. Guidelines in practice: instrument cleaning. AORN J. 2021;114(3):241-251. doi: 10.1002/aorn.13495.

^[4]^National Health and Family Planning Commission of the People’s Republic of China. Central sterile supply department (CSSD)-Part 2: Standard for operating procedure of cleaning, disinfection and sterilization. Chinese Journal of Infection Control. 2017;16(10):986-992.

^[5]^Rutala WA, Weber DJ; Society for Healthcare Epidemiology of America. Guideline for disinfection and sterilization of prion-contaminated medical instruments. Infect Control Hosp Epidemiol. 2010;31(2):107-117. doi: 10.1086/650197.

^[6]^Department of Health. Health Technical Memorandum 01 -01: Decontamination of reusable medical devices [EB/OL]. [2022-06-211. https://www.england.nhs.uk/publication/decontaminationof-surgicakinstruments-htm-01-01.

^[7]^Chinese Nursing Association. T/CNAS 09-2019 Cleaning technique for medical instruments. Beijing: Standards Press of China, 2024.

# ^[8]^Centers for Disease Control and Prevention, Guideline for disinfection and sterilization in healthcare facilities (2008) [EB/OL]. [2022-06-21]. https://www. cdc. gov/infectioncontrol/guidelines/disinfection.

# ^[9]^Commission for Hospital Hygiene and Infection Prevention (KRINKO); Federal Institute for Drugs and Medical Devices (BfArM). Anforderungen an die Hygiene bei der Aufbereitung von Medizinprodukten. Empfehlung der Kommission für Krankenhaushygiene und Infektionsprävention (KRINKO) beim Robert Koch-Institut (RKI) und des Bundesinstitutes für Arzneimittel und Medizinprodukte (BfArM) [Hygiene requirements for the reprocessing of medical devices. Recommendation of the Commission for Hospital Hygiene and Infection Prevention (KRINKO) at the Robert Koch Institute (RKI) and the Federal Institute for Drugs and Medical Devices (BfArM)]. Bundesgesundheitsblatt Gesundheitsforschung Gesundheitsschutz. 2012;55(10):1244-310. German. doi: 10.1007/s00103-012-1548-6. .

^[10]^ASGE Quality Assurance in Endoscopy Committee; Calderwood AH, Day LW, Muthusamy VR, Collins J, Hambrick RD 3rd, Brock AS, Guda NM, Buscaglia JM, Petersen BT, Buttar NS, Khanna LG, Kushnir VM, Repaka A, Villa NA, Eisen GM. ASGE guideline for infection control during GI endoscopy. Gastrointest Endosc. 2018;87(5):1167-1179. doi: 10.1016/j.gie.2017.12.009.

^[11]^Gastroenterological Society of Australia. Infection prevention and control in endoscopy 2021[EB/OL].[2025-03-21]. https://www.gesa.org.au/public/13/files/Education%20%26%20Resources/Clinical%20Practice%20Resources/Endoscopy%20Infection%20Control/Infection%20Prevention%20and%20Control%20in%20Endoscopy.pdf.

^[12]^SPEER T,ALFA M,COWEN A,et al. Endoscope disinfection update:A guide to resource sensitive reprocessing[S/OL].Australia:World Gastroenterology Organisation Melbourne,2019.[2022-06-21].https://WGO Practice Guidelines style template(world gastroenterology. org).

^[13]^Beilenhoff U, Biering H, Blum R, Brljak J, Cimbro M, Dumonceau JM, Hassan C, Jung M, Kampf B, Neumann C, Pietsch M, Pineau L, Ponchon T, Rejchrt S, Rey JF, Schmidt V, Tillett J, van Hooft JE. Reprocessing of flexible endoscopes and endoscopic accessories used in gastrointestinal endoscopy: Position Statement of the European Society of Gastrointestinal Endoscopy (ESGE) and European Society of Gastroenterology Nurses and Associates (ESGENA) - Update 2018. Endoscopy. 2018;50(12):1205-1234. doi: 10.1055/a-0759-1629.

^[14]^Meyer GE, Preventing infection transmitted by gastrointestinal endoscopy [EB/OL].(2024-04-30)[2025-04-01]. https://www.uptodate.cn/contents/zh-Hans/preventing-infection-transmitted-by-gastrointestinal-endoscopy?search=Preventing%20infection%20transmitted%20by%20gastrointestinal%20endoscopy&source=search_result&selectedTitle=1~150&usage_type=default&display_rank=1.

^[15]^Liu YX, Xing YB, Gong YX. Regulation for cleaning and disinfection technique of flexible endoscope WS507-2016. Chinese Journal of Infection Control. 2017;16(6):587-592.

^[16]^Darbord JC. Importance of cleaning for reprocessing endoscopes and thermolabile sterile medical devices: French use and regulations. J Hosp Infect. 2004;56 Suppl 2:S40-43. doi: 10.1016/j.jhin.2003.12.028.

^[17]^Department of Health, Health Technical Memorandum 01-06 :Decontamination of flexible endoscopes[EB/OL]. [2022-06-21]. https://www.england.nhs.uk/publication/management-and-decontamination-of-flexible-endoscopes-htm-01-06/.

^[18]^Loyola M, Babb E, Bocian S, Diskey A, Friis CM, Granato A, Schmit A, Selking S; SGNA Practice Committee 2017-18. STANDARDS OF INFECTION PREVENTION IN REPROCESSING FLEXIBLE GASTROINTESTINAL ENDOSCOPES. Gastroenterol Nurs. 2020;43(3):E142-E158. doi: 10.1097/SGA.0000000000000536. .

^[19]^Ling ML, Ching P, Cheng J, Lang L, Liberali S, Poon P, Shin Y, Sim C. APSIC dental infection prevention and control (IPC) guidelines. Antimicrob Resist Infect Control. 2023;12(1):53. doi: 10.1186/s13756-023-01252-w.

^[20]^National Health and Family Planning Commission of the People’s Republic of China.Regulation for disinfection and sterilization technique of dental instruments WS 506-2016. Chinese Journal of Infection Control. 2017;16(8):784-792.

^[21]^Department of Health. Health Technical Memorandum 01-05:Decontamination in primary care dental practices [EB/OL].[2022-06-21]. https://www.england.nhs.uk/publication/decontamination-in-primary-care-dental-practices-htm-01-05.

^[22]^Chang DF, Mamalis N; Ophthalmic Instrument Cleaning and Sterilization Task Force. Guidelines for the cleaning and sterilization of intraocular surgical instruments. J Cataract Refract Surg. 2018;44(6):765-773. doi: 10.1016/j.jcrs.2018.05.001.

^[23]^Ophthalmologist Branch of the Chinese Medical Doctor Association, Hospital Infection Control Committee of the Chinese Preventive Medicine Association, Disinfection Branch of the Chinese Preventive Medicine Association, et al. Guidelines for Ophthalmic Surgery Management, Infection Control, and Disinfection and Sterilization in China(I). Chinese Journal of Ophthalmology. 2016;52(3):167-173.

^[24]^Ophthalmic Nursing Committee of the Chinese Nursing Association, The Vitreoretinal Disease Group of the Ophthalmology Branch of the Chinese Medical Association. Expert Consensus on Cleaning Methods for Ophthalmic Surgical Instruments Attached to Silicone Oil (2020). Chinese Journal of Optometry & Ophthalmology and Visual Science. 2020;22(7):481-484.

^[25]^Chinese Research Hospital Association. T/CRHA 079—2024 Practice specification for pretreatment of reusable medical devices. Beijing: Standards Press of China, 2024.

^[26]^Cleaning and Disinfection Group of the Digestive Endoscopy Branch of the Chinese Medical Association. Interpretation of Chinese expert consensus on gastrointestinal endoscope reprocessing (2024, Chongqing). Chinese Journal of Digestive Endoscopy. 2024;41(09):673-684.
