# Supplementary material for: Evidence-based preprocessing improvement bundle for surgical instruments: reducing surgical infection risks via quality improvement
Source: Front Public Health. 2026 Oct 27;14:1930642. doi: 10.3389/fpubh.2026.1930642 (PMC13761332; doi:10.3389/fpubh.2026.1930642)
Supplement: Supplementary file 2 [file Supplementary_file_2.docx]

**Supplementary file 2** Audit checklist for the 20 audit criteria for surgical instrument preprocessing

| Criterion number | Content | Brief definition and key points for audit | Audit criteria (Y/N) | Audit results (Y/N) |
| --- | --- | --- | --- | --- |
| 1 | After surgery, nurses or workers remove visible bloodstains, dirt and human tissues from the surfaces of surgical instruments. | The quality controllers observe whether visible bloodstains, dirt, and human tissues have been removed from the instruments after surgery. | Y: Visible bloodstains, dirt, and human tissues have been removed from the surfaces of the instruments.  N: Visible bloodstains, dirt, or human tissues remain on the surfaces of the instruments. |  |
| 2 | Enzyme-based preprocessing agents, including gels, foams, and sprays, or a wet cloth are used to keep instruments moist. | Whether instruments are kept moist using enzyme-based preprocessing agents, including gels, foams, and sprays, or a wet cloth. | Y: Instruments are kept moist using enzyme-based preprocessing agents or a wet cloth.  N: Instruments were not kept moist. |  |
| 3 | After each use, the flexible endoscope surface is wiped with a lint-free cloth while the endoscope remains connected to the light source. | Whether the endoscope surface is wiped with a lint-free cloth while the endoscope remains connected to the light source after surgery. | Y: The endoscope surface is wiped with a lint-free cloth while the endoscope remains connected to the light source.  N: Wiping is not performed or is performed only after disconnection from the light source. |  |
| 4 | The insertion portion of the flexible endoscope is immersed into a container containing a cleaning agent; the lumen is continuously flushed until no visible contaminants are seen in the fluid flowing from the lumen; visual inspections are performed on the surface of the flexible endoscope to identify any indentations or damage. | The lumen of the insertion portion of the flexible endoscope is continuously flushed, and visual inspections are performed on the surface of the flexible endoscope. | Y: The lumen is continuously flushed until no visible contaminants are seen in the fluid flowing from the lumen, and visual inspections of endoscope surfaces are performed.  N: Lumen is inadequately flushed, or visual inspections are not performed. |  |
| 5 | The light source connector is removed from the flexible endoscope, a water-resistant cap is attached to the connector, and the endoscope cable surface is wiped. | The light source connector is removed from the flexible endoscope before a water-resistant cap is attached to the connector and the endoscope cable surface is wiped. | Y: A water-resistant cap is attached to the connector and the endoscope cable surface is wiped.  N: The water-resistant cap is not attached to the connector, or endoscope cable surface is not wiped. |  |
| 6 | After use, rigid endoscopes with lumens are disassembled according to their structure. The inner cores are removed, and the lumens are flushed. | The lumened instruments are disassembled; the inner cores are removed; and the lumens are flushed. | Y: The lumened instruments are disassembled; the inner cores are removed; and the lumens are flushed.  N: The lumened instruments are not disassembled, or the lumens are not flushed. |  |
| 7 | Dental handpieces, electric scalers and electrosurgical units are stored in dry collection containers. | Whether the collection containers used for the instruments are dry. | Y: The instruments are stored in dry collection containers.  N: The collection containers are not dry. |  |
| 8 | Ophthalmic instruments are immersed in sterile water immediately after viscoelastic agents are used. | Immersion procedure for ophthalmic instruments after viscoelastic agents are used. | Y: Ophthalmic instruments are immersed in sterile water immediately after surgery.  N: No immersion procedure is performed on ophthalmic instruments, or the instruments are not immersed in sterile water. |  |
| 9 | Intraocular surgical instruments are rinsed, wiped, and preprocessed using deionized water (with a bacterial concentration <10 cfu/mL). | Deionized water (with a bacterial concentration <10 cfu/mL) is used to preprocess intraocular surgical instruments. | Y: The instruments are preprocessed using liquid that conforms to the requirements.  N: The instruments are preprocessed using liquid that does not conform to the requirements, or are not preprocessed. |  |
| 10 | Ophthalmic instruments are placed in their dedicated collection containers. | Whether dedicated collection containers are provided for ophthalmic instruments. | Y: Ophthalmic instruments are placed in their dedicated collection containers.  N: Ophthalmic instruments are mixed with non-ophthalmic instruments. |  |
| 11 | Disposable instruments, such as blades and sutures, are discarded at the point of use. | Dispose of disposable instruments, such as blades and sutures. | Y: Disposable instruments are discarded after use.  N: Disposable instruments are mixed with reusable instrument. |  |
| 12 | Surgical instruments are transported to the CSSD decontamination area within one hour after preprocessing. Delicate surgical instruments must be placed in containers with fixture slots or silicone mats for transportation. | Instrument transport timeliness and delicate instrument protection. | Y: Instruments are transported to the CSSD decontamination area within one hour after preprocessing. Delicate instruments are protected against collision.  N: Transport takes longer than one hour, or no collision prevention measures were adopted to protect the delicate instruments. |  |
| 13 | Flexible endoscopes are placed in dedicated collection containers, while rigid endoscopes are placed in containers with fixture slots. | Choices of collection containers for endoscopic instruments. | Y: Endoscopic instruments are placed in dedicated collection containers or those with fixture slots.  N: The collection containers do not meet the requirements. |  |
| 14 | Surgical instruments are placed in sealed containers, such as a cabinet with a lid, during transportation, clearly labeled with the type of contamination. The external surfaces of the transport containers remain uncontaminated. | Sealed containers, labeling, and container surfaces remaining clean | Y: Instruments are placed in sealed containers during transportation, clearly labeled with the type of contamination. The external surfaces of the transport containers remain uncontaminated.  N: The transport containers do not meet the requirements; the containers are unlabeled; or the external surfaces of the transport containers are contaminated. |  |
| 15 | CSSD staff sort instruments at the decontamination area according to their degree of contamination, precision, and structures, and then disassemble the instruments. Severely contaminated instruments are soaked and scrubbed before cleaning. | Instruments are sorted and disassembled at the CSSD decontamination area. Severely contaminated instruments are soaked and scrubbed before cleaning. | Y: Instruments are sorted and disassembled. Severely contaminated instruments are soaked and scrubbed before cleaning.  N: Instruments are not sorted or disassembled. Severely contaminated instruments are not soaked or scrubbed before cleaning. |  |
| 16 | CSSD staff remove all accessories from the flexible endoscopes prior to cleaning, inspect endoscope surfaces for indentations and damage, and perform a leak test. | All accessories are removed from the endoscopes before cleaning. Endoscope surfaces are inspected, and a leak test is performed. | Y: All accessories are removed from the flexible endoscopes before cleaning. A leak test is performed, and endoscope surfaces are inspected.  N: Not all accessories are removed from the flexible endoscopes before cleaning; no leak test is performed; or endoscope surfaces are not inspected. |  |
| 17 | All cleaning tools for preprocessing are for single use only. | Cleaning tools for preprocessing are for single use only. | Y: Cleaning tools for preprocessing are for single use only and are discarded after use.  N: Cleaning tools are reused. |  |
| 18 | Cleaning agents or water used for lumen flushing are for single use only. | Cleaning agents or water are for single use only. | Y: Cleaning agents or water are for single use only and are discarded after use.  N: Cleaning agents or water are reused. |  |
| 19 | All preprocessing personnel have received relevant training and passed assessments of preprocessing knowledge and skills. | Staff training and assessment | Y: Training is provided and preprocessing personnel have passed assessments.  N: No training is provided, or preprocessing personnel have not passed assessments. |  |
| 20 | Four preprocessing training sessions are conducted annually, each followed by an assessment and documentation. Routine quality control includes random checks of preprocessing knowledge and assessments of practical skills. | Quality control and documentation for training | Y: At least four training sessions are conducted annually, each followed by an assessment and documentation.  N: Training is conducted less frequently than required or relevant records are incomplete or unavailable. |  |
