# Supplementary material for: Evidence-based preprocessing improvement bundle for surgical instruments: reducing surgical infection risks via quality improvement
Source: Front Public Health. 2026 Oct 27;14:1930642. doi: 10.3389/fpubh.2026.1930642 (PMC13761332; doi:10.3389/fpubh.2026.1930642)
Supplement: Supplementary file 3 [file Supplementary_file_3.docx]

**Supplementary file 3** Assessment of surgical instrument preprocessing knowledge

Dear colleagues,

Thank you for taking the time to complete the assessment. This assessment aims to investigate your mastery of surgical instrument preprocessing knowledge. This is an anonymous assessment. Data collected will be used for research only and will be kept strictly confidential. Please understand your participation is completely voluntary and you have the right to withdraw your consent or discontinue participation at any time. Please answer each question honestly. By answering the questions and submitting your answers, you are considered to have agreed to participate in this study. The assessment comprises two parts. Part I is demographic information and Part II contains the questions about surgical instrument preprocessing knowledge. Please read the instructions and each question carefully. Your responses are greatly appreciated. Please complete the survey independently and do not leave any questions unanswered.

Thank you for your participation.

Part 1. Demographic information

1. What is your gender? [Single-choice question]*

○A. Male

○B. Female

2. What is your age? [Please fill in the blank]*

_________________________________

3. Length of your work experience: [Please fill in the blank]*

_________________________________

4. What is your highest educational attainment? [Single-choice question]*

○A. Secondary vocational school or below

○B. Assoiate degree

○C. Bachelor’s degree

○D. Master’s degree or above

5.What is your professional title? [Single-choice question]*

○A. Nurse (entry-level)

○B. Senior Nurse (mid-level)

○C. Charge Nurse (senior)

○D. Deputy Chief Nurse or above

○E. Other _________________

6.What is your current job role? [Multiple-choice question]*

○A. Staff nurse

○B. Teaching team leader

○C. Quality control nurse

○D. Head nurse

○E. Other _________________

Part 2. Knowledge of surgical instrument preprocessing

After use, the best golden window period for preprocessing at the point of use is ( ) [Single-choice question]*

○A. 15 minutes to 1 hour

○B. 2 to 4 hours

○C. 4 to 6 hours

○D. 24 hours

2. Which of the following products are strictly prohibited for use in instrument preprocessing? (Multiple-choice question)*

○A. Saline solution (normal saline)

○B. Aldehyde disinfectants

○C. Peracetic acid

○D. Multi-enzyme preprocessing foam

3. In terms of time limit requirement for endoscope preprocessing at the point of use, how long will it take for endoscope to be transported to the central sterile supply department (CSSD)? [Single-choice question]*

○A. Within 15 minutes

○B. Within 1 hour

○C. Within 4 hours

○D. Within 6 hours

4. In terms of benchmark requirements for flushing the lumened endoscopes, which of the following statements is correct ( ) [Single-choice question]*

○A. 100-150ml of fluid, and continuous flushing for 5-10 seconds.

○B. 200-250ml of fluid or continuous flushing for 10-20 seconds until no visible contaminants are seen in the fluid flowing from the lumens.

○C. Only flush the external surface; preprocessing is not required for the interior surface of the lumen.

○D. Repeatedly flush the lumen with normal saline.

5. Which of the following statements about the preprocessing of complex-structured oral instruments (dental handpieces and curettes) are correct? [Multiple-choice question]*

○A. They should be kept moist and placed in sealed collection containers.

○B. Soft water or enzyme preprocessing agents can be used as the moistening agents.

○C. They can be directly dried and stored without moistening.

○D. They can be transported together with other surgical instruments.

6. The correct storage method for dental handpieces and electric scalers after preprocessing is ( ) [Single-choice question]*

○A. Soaked in multi-enzyme solution for moisture retention and storage.

○B. Stored in dry collection containers after initial decontamination.

○C. Wrapped in a damp gauze and placed openly.

○D. Sealed and stored after infusing with sterile water.

7. Preprocessing requirements for intraocular instruments include: [Multiple-choice question]*

○A. Preferably use distilled water or deionized water (with a bacterial concentration < 10 cfu/mL).

○B. Enzyme cleaners are not routinely recommended.

○C. Keep separate from non-ophthalmic instruments; they are not mixed with other instruments during loading.

○D. Instruments are immersed in sterile water immediately after viscoelastic agents are used.

8. The requirements for the transportation of contaminated instruments include ( ) [Multiple-choice question]*

○A. The instruments are placed in a container with a lid, labeled with the type of contamination.

○B. Avoid passing through public areas and sterile item storage areas.

○C. Collision prevention measures are adopted to protect delicate instruments.

○D. Endoscopes are mixed with other instruments for transportation to save space.

9. The requirement for the preprocessing and disposal of instruments contaminated with prions is ( ) [Single-choice question]*

○A. Treated as ordinary contaminated instruments.

○B. Keep moist, double-layer sealed packaging, labeled with the type of contamination, and collected by CSSD staff.

○C. Instrument moistening is not required. The instruments directly undergo high-temperature disinfection.

○D. Transported and reprocessed together with other instruments.

10. The requirements for the management of instrument preprocessing supplies and personnel include ( ) [Multiple-choice question]*

○A. Preprocessing brushes and cloths are recommended for single use and should be discarded after use.

○B. Water and cleaning agents for lumen flushing are for single use only.

○C. Preprocessing personnel have receive professional training and possess the necessary competence.

○D. Regular training and assessment on instructions for use, preprocessing procedures and personal protective measures are conducted.
